# Identification of a Developmental Switch in Information Transfer between Whisker S1 and S2 Cortex in Mice

**DOI:** 10.1523/JNEUROSCI.2246-21.2022

**Published:** 2022-06-01

**Authors:** Linbi Cai, Jenq-Wei Yang, Chia-Fang Wang, Shen-Ju Chou, Heiko J. Luhmann, Theofanis Karayannis

**Affiliations:** ^1^Laboratory of Neural Circuit Assembly, Brain Research Institute, University of Zürich, CH-8057, Zürich, Switzerland; ^2^Institute of Physiology, University Medical Center, Johannes Gutenberg University Mainz, 55128, Mainz, Germany; ^3^Institute of Cellular and Organismic Biology, Academia Sinica, Taipei, 11529, Taiwan

**Keywords:** cortex, development, primary, secondary, somatosensory, whisker

## Abstract

The whiskers of rodents are a key sensory organ that provides critical tactile information for animal navigation and object exploration throughout life. Previous work has explored the developmental sensory-driven activation of the primary sensory cortex processing whisker information (wS1), also called barrel cortex. This body of work has shown that the barrel cortex is already activated by sensory stimuli during the first postnatal week. However, it is currently unknown when over the course of development these stimuli begin being processed by higher-order cortical areas, such as secondary whisker somatosensory area (wS2). Here we investigate the developmental engagement of wS2 by whisker stimuli and the emergence of corticocortical communication from wS1 to wS2. Using *in vivo* wide-field imaging and multielectrode recordings in control and conditional KO mice of either sex with thalamocortical innervation defects, we find that wS1 and wS2 are able to process bottom-up information coming from the thalamus from birth. We also identify that it is only at the end of the first postnatal week that wS1 begins to provide functional excitation into wS2, switching to more inhibitory actions after the second postnatal week. Therefore, we have uncovered a developmental window when information transfer between wS1 and wS2 reaches mature function.

**SIGNIFICANCE STATEMENT** At the end of the first postnatal week, the primary whisker somatosensory area starts providing excitatory input to the secondary whisker somatosensory area 2. This excitatory drive weakens during the second postnatal week and switches to inhibition in the adult.

## Introduction

Rodents are born with immature sensory systems (Khazipov et al., 2004; Leighton and Lohmann, 2016). The postnatal development and maturation of brain circuits are essential for the detailed representation of environmental stimuli, which enables animals to interact with the external world in a refined manner (Erzurumlu and Gaspar, 2012; Feldmeyer et al., 2013; Petersen, 2019). The somatosensory whisker system is key for rodent navigation and exploration of tactile stimuli already at birth (Akhmetshina et al., 2016; Yang et al., 2018). The information coming from the whiskers is conveyed via the trigeminal brainstem nuclei to the primary somatosensory thalamic nucleus, the ventro-posterior-medial nucleus (VPM), as well as the higher-order medial thalamic part of the posterior nucleus (POm). This information is then fed forward to the “barrels” of the primary whisker somatosensory cortex (wS1), as well as the secondary whisker somatosensory cortex (wS2) and the motor cortex (Goebbels et al., 2006; Minlebaev et al., 2011; Yamashita et al., 2013; Yamashita and Petersen, 2016; Staiger and Petersen, 2021). Anatomical studies have demonstrated dense reciprocal connections between wS1 and wS2 (Kwon et al., 2016; Minamisawa et al., 2018), and it has also been functionally shown that wS1 and wS2 represent whisker stimuli in a mirror-like topographic manner (Adibi, 2019; Hubatz et al., 2020). In contrast to wS1, wS2 is preferentially activated by simultaneous whisker stimuli or single whisker stimuli delivered at high frequency (Menzel and Barth, 2005). These findings, together with a shorter delay in arrival of sensory stimuli in wS1 compared with wS2 and larger receptive fields in wS2 versus wS1, have suggested that wS2 is a higher-order brain region that would process information coming from wS1. In this respect, the functional interaction between wS1 and wS2 has been studied in the behaving adult rodent, and coordinated activity from wS1 to wS2 has been shown to be essential for proper whisker-associated perception and learning behavior. For instance, wS2-projecting wS1 neurons show touch-related responses during a texture discrimination task (Chen et al., 2013), and they also develop specific patterns of activity during learning the task (Chen et al., 2015). Furthermore, wS2-projecting wS1 neurons have also been found to be involved in the goal-directed sensorimotor transformation of a whisker touch to licking motor output (Yamashita and Petersen, 2016) and show higher choice-related activity than other neurons in layer 2/3 (Kwon et al., 2016). Although previous studies addressed the development of sensorimotor processing (Khazipov et al., 2004; Dooley and Blumberg, 2018; Gómez et al., 2021), little is known about the developmental stage when wS2 begins processing sensory information coming directly from the thalamus and/or indirectly via wS1. Revealing this would signify the beginning of higher-order representation of tactile stimuli in mammals. One informed hypothesis is that this occurs at the end of the second postnatal week, when mice begin to whisk actively and increase their locomotor activity searching for tactile information (Landers and Zeigler, 2006; Arakawa and Erzurumlu, 2015).

Here, we have used a variety of *in vivo* approaches to assess the engagement of wS2 in whisker stimuli and probe its feedforward activation by wS1 during development. By simultaneously recording wS1 and wS2 activity *in vivo* using either wide-field imaging or silicon probes, we find that wS2 processes sensory inputs from the first few days of postnatal life, activated directly through thalamocortical projections. It is only at the end of the first postnatal week that a long-lasting sensory-driven activity in wS1 starts propagating to wS2 driving its latent spiking phase, as shown through acute pharmacological manipulation of activity and genetic disruption of thalamocortical transmission. This excitatory drive of wS1 to wS2 weakens by the end of the second postnatal week, and switches to inhibition in the adult. Thus, our work has identified a developmental window of information transfer from wS1 to wS2, providing insights into the emergence of higher-order representation of the environment in the cortex.

## Materials and Methods

### Animals

Animal experiments were approved by the Cantonal Veterinary Office Zurich, the local German ethics committee (#23177-07/G10-1-010), and the Academia Sinica Institutional Animal Care and Use Committee in Taiwan. Animals were in husbandry with a 12 h reverse darklight cycle (7:00 A.M. to 7:00 P.M. dark) at 24°C and variable humidity. Voltage-sensitive dye imaging (VSDI) was performed on C57BL6J mice of either sex. *Lhx2* conditional KO (cKO, *Lhx2*^f/f^; *Nex*-Cre) mice and *TCA-GFP* mice were generated as described (Goebbels et al., 2006; Chou et al., 2009; Suzuki and Bekkers, 2011; Mizuno et al., 2014). All lines were maintained on a C57BL/6 background. Wide-field imaging and multielectrode recordings were performed on Snap25-2A-GCaMP6s-D mice of either sex.

### Animal surgery

We used 77 Snap25-GCaMP6s mice at the ages from P0 to P56 for wide-field imaging and multielectrode recordings. Mice were light anesthetized by urethane (the final concentration is 1 g/1 kg mouse weight for P1-P8 mice, 1-1.25 g/1 kg for P14-P16 mice, and 1.5 g/1 kg for P25-P56 mice) throughout the whole experiment. A heating pad was used to maintain the mouse body temperature at 37°C. The depth of anesthesia was checked with breathing rate and paw reflexes throughout the experiment. If the respiratory rate was slower than 3 times every 2 s, as measured by eye, the mouse would be considered as deeply anesthetized and would be excluded from the analysis.

The skull of the right hemisphere was exposed by removing the skin on top, and a metallic head holder was implanted on the skull with cyanoacrylate glue and dental cement. A 20G needle was used to open ∼2 mm × 2 mm cranial window which exposed both S1 and S2. Extreme care was taken not to cause damage or surface bleeding in neonatal pups.

### Whisker stimulation

A single whisker was stimulated 1 mm from the snout in a rostral to caudal direction (∼1 mm displacement) using a stainless-steel rod (1 mm diameter) connected to a miniature solenoid actuator. The movement of the tip of the stimulator bar was measured precisely using a laser micrometer (MX series, Metralight) with a 2500 Hz sampling rate. The stimulus takes 26 ms to reach the maximal 1 mm whisker displacement, with a total duration of 60 ms until it reaches baseline (Yang et al., 2017).

### VSDI

The procedure of VSDI was according to our previous report with small modifications (Yang et al., 2013; Luhmann, 2017). Briefly, the VSD RH1691 or RH2080 (Optical Imaging) was dissolved at 1 mg/ml in Ringer's solution containing the following (in mm): 135 NaCl, 5.4 KCl, 1 MgCl_2_, 1.8 CaCl_2_, and 5 HEPES (pH was set to 7.2 with NaOH). The VSD was topically applied to the surface of the opened skull and allowed to diffuse into the cortex for 40-60 min. Subsequently, the unbound dye was carefully washed away with Ringer's solution. The cortex was covered with a 1% low-melting agarose, and a coverslip was placed on top to stabilize the tissue. Imaging was performed using a MiCam Ultima or MiCAM05 high-speed camera (Scimedia) with 625 nm excitation and 660 nm long-pass filtered emission. The FOV was 3.1 × 3.1 mm^2^ with 100 × 100 pixels for recordings using the MiCam Ultima and 6.8 × 6.8 mm^2^ with 256 × 256 pixels for recordings using the MiCAM05. The frame sampling frequency was 500 Hz. The evoked activity following whisker stimulation was averaged from 5 recording sessions.

### Wide-field calcium imaging

The principal whisker-related S1 barrel columns (B1, B2, C1, and C2) and their corresponding representations in S2 were identified by wide-field calcium imaging. Before P16, the cortical surface was visualized through the intact skull. In mice older than P21, the skull was thinned to have better visualization of the calcium signal during wide-field imaging. By shining blue light (488 nm LED) on the cortical surface, the functional maps in S1 and S2 were revealed by stimulating the principal whiskers. Images were acquired through a 1× Nikon objective or 1.25× Olympus objective with a CCD camera with 5 or 10 fps. The recordings lasted 10 s with a 2 s baseline and 8 s poststimulation period. The wide-field calcium signals obtained for principal barrels were then mapped to the blood vessel reference image and used to guide the location of the craniotomy and subsequent *in vivo* multielectrode recording.

### In vivo multielectrode recordings

S1 and S2 neural activity was recorded simultaneously with a 64-channel silicon probe inserted perpendicularly into the cortex. Each of the 8 shanks has 8 recording sites (100 µm apart). The distance between each shank is 200 µm (NeuroNexus Technologies). Insertion of the silicon probe was guided by the wide-field calcium imaging results. The specific principal barrel location in wS1 and the corresponding activated wS2 area were identified with wide-field imaging before electrode insertion. Subsequently, using the blood vessel pattern as a reference, the silicon probe was guided and inserted. A silver wire was placed into the cerebellum as a ground electrode. Before insertion, the silicon probe was dipped into DiI solution; therefore, the insertion points were marked after removing the probe. Together with the wide-field results, this allowed us to confirm *post hoc* that the recordings were performed in the specific barrel and the corresponding wS2. All data were acquired at 20 kHz and stored with MC_RACK software (Multi Channel Systems). The total duration of multielectrode recordings varied between 3 and 5 h.

For the spiking blockade experiments, after a capillary containing 2 μm TTX was inserted into wS1 without injecting TTX into the cortex, 20 trails of single, and 10 Hz whisker stimulations were applied and S1 and S2 activity was recorded. Afterward, 200 nl of TTX in the capillary were injected into the cortex to block activity in wS1. The same stimulation paradigm was performed again, and wS1 and wS2 activity was recorded.

### Perfusion and immunostaining

WT *(Lhx2f/f:TCA-GFP)* and *cKO (Lhx2f/f:NexCre:TCA-GFP)* mice were transcardially perfused with 4% phosphate-buffered PFA at P7, and then postfixed in the same solution for 1 d. Brains were then cryoprotected with 30% sucrose in PBS, embedded in Tissue Tek OCT compound (Sakura Finetek), and cut in 20-25 µm sections on a cryostat (Leica). Coronal sections were immunostained with rabbit anti-GFP antibody (Millipore) and Alexa-conjugated secondary antibodies (Jackson ImmunoResearch Laboratories). Fluorescent images were acquired using a LSM880 confocal microscope (Zeiss) with the scaling of 0.647 µm × 0.647 µm per pixel.

For VGlut2 immunohistochemistry, after the brain was perfused with PBS, fixed with 4% PFA and cryoprotected with 30% sucrose in PBS as described above, it was cut parallel to the surface at 300-μm-thick sections using a vibratome. Subsequently, rabbit anti-VGlut2 antibody (Synaptic Systems) (1:500) and Alexa-conjugated secondary antibodies (Thermo Fisher Scientific) (1:1000) were applied on free-floating sections. The slices were incubated for 2 d in the primary antibody at 4°C and after thorough washing with PBS, for 4 h in the secondary antibody at room temperature. They were then mounted on slides and covered with Fluoromount medium (Thermo Fisher Scientific) before coverslipped and imaged at a confocal microscope.

### Experimental design and statistical analysis

#### Analysis of VSDI data

**Figure 1.**
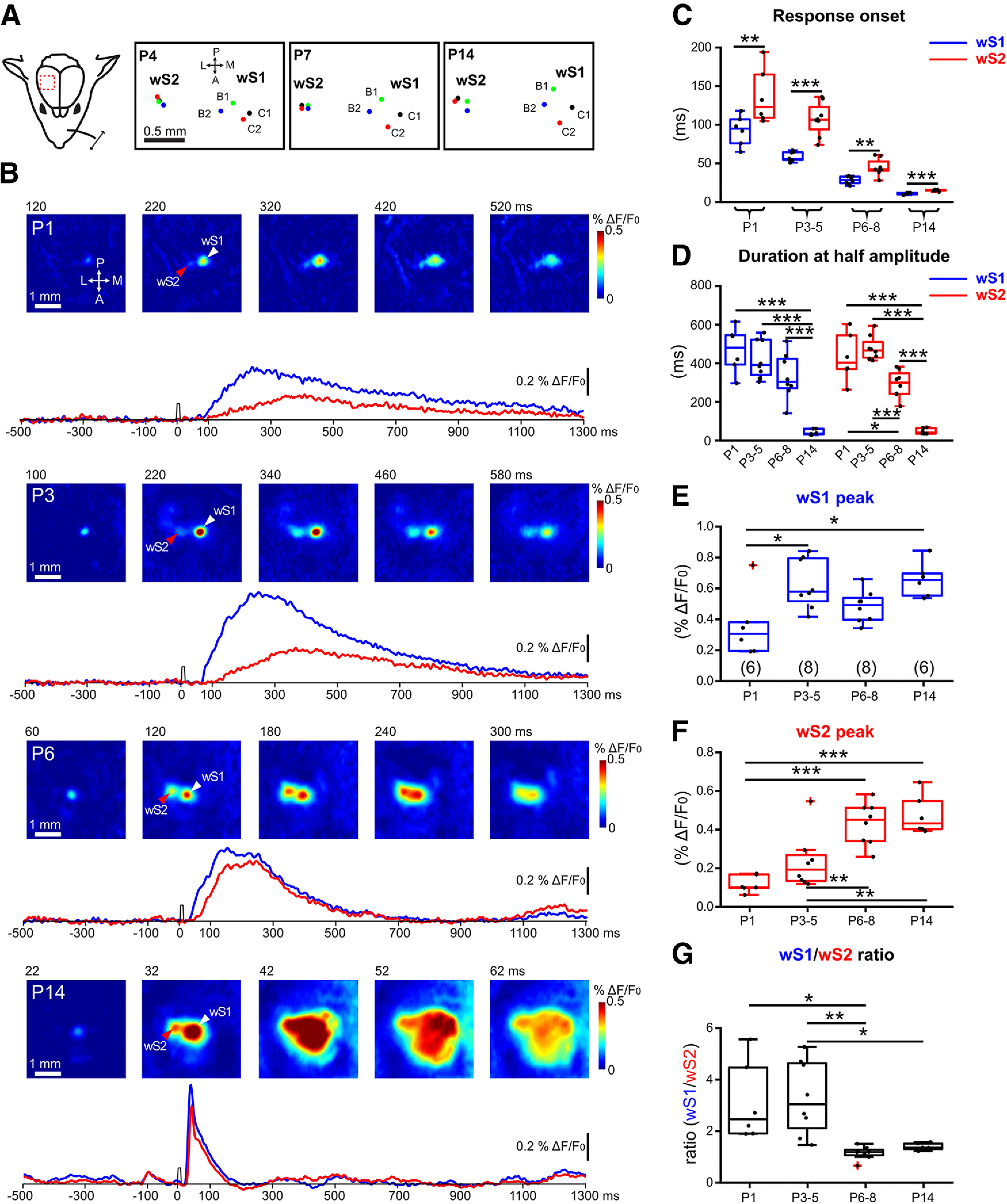
Developmental changes of sensory-evoked VSDI responses in wS1 and wS2. ***A***, Left, Schematic illustration of the experimental design. A single whisker deflection is performed together with VSDI of the whisker somatosensory cortex. Right, The spatial location of the representation of activity for four whiskers (B1, B2, C1, and C2) in wS1 and wS2 in a P4, P7, and P14 mouse. ***B***, VSDI of responses elicited by B3-whisker deflection in different age groups of WT mice (P1, P3, P6, and P14). White arrowhead indicates the center of the wS1 response. Red arrowhead indicates the center of the wS2 response. Bottom row, 1.8-s-long optical recording traces obtained by analyzing the signal in the centers of wS1 and wS2 over time (blue trace for wS1 and red trace for wS2). Time point of whisker stimulation at 0 ms. ***C***, The onset times of evoked responses in wS1 and wS2 in the different age groups. Asterisks indicate significant difference between wS1 and wS2 using a paired *t* test. P1: *p* = 0.0041, *t* = 5.007; P3-P5: *p* = 0.0001, *t* = 7.631; P6-P8: *p* = 0.0015, *t* = 5.063; *p* = 0.0005, *t* = 8.032. ***D***, The duration at the half-maximal amplitude of VSDI for different age groups in wS1 and wS2. Statistics: one-way ANOVA with Bonferroni's multiple comparison test. S1: *p* < 0.0001; S2: *p* < 0.0001. ***E***, Box plot represents the evoked peak amplitude in the center of wS1 in the different age groups (*n* = 6 barrels from *N* = 3 P1 mice; *n* = 8 barrels from *N* = 4 P3-P4 mice; *n* = 8 barrels from *N* = 4 P6-P8 mice; *n* = 6 barrels from *N* = 3 P14 mice). Statistics: one-way ANOVA with Bonferroni's multiple comparison test. *p* = 0.0044. ***F***, Box plot represents the evoked peak amplitude in the center of wS2 in the different age groups. Statistics: one-way ANOVA with Bonferroni's multiple comparison test. *p* < 0.0001. ***G***, Box plot represents the evoked peak amplitude in wS1 divided by the evoked peak amplitude in wS2 in the different age groups. Statistics: ANOVA with Bonferroni's multiple comparison test. *p* = 0.0006. *p < 0.05; **p < 0.01; ***p < 0.001. All the values of data points and the mean and SEM of each group are in Extended Data Figure 1-1.

Fluorescence signals were analyzed using custom-made routines in MATLAB (The MathWorks). The fluorescence change (ΔF/F_0_) was calculated as the change of fluorescence intensity (ΔF) in each pixel divided by the initial fluorescence intensity (F_0_) in the same pixel. The center of a single barrel was functionally determined by the local maxima of the initial appearance of the VSDI response (see Fig. 1*A*). The fluorescence signals in the functional center of the barrel were used to analyze the peak amplitude, and onset time. The onset time was detected by the threshold in 1-fold baseline SD.

#### Analysis of wide-field calcium imaging data

Wide-field calcium imaging data were analyzed using a custom-made MATLAB script (2019a, The MathWorks). The fluorescence change (ΔF/F_0_) was calculated as the change of fluorescence intensity (ΔF) in each pixel divided by the baseline fluorescence intensity (F_0_, average of 500 ms absolute fluorescence before the onset of whisker deflection) in the same pixel. The S1 and S2 evoked activity over different age group was compared by using the highest evoked calcium response.

#### Analysis of *in vivo* multielectrode silicon probe data

Extracellular silicon probe data were analyzed using a custom-made MATLAB script (2019a, The MathWorks). The raw data signal was bandpass filtered (0.8-5 kHz) and the multiunit activity (MUA) was extracted with the threshold of 5 times the SD of baseline. The current source density (CSD) map was used to identify L2-3, L4, and L5. The earliest CSD sink was identified as layer 4, followed by L2-3 and L5 (Reyes-Puerta et al., 2015; van der Bourg et al., 2017). The MUAs were separated, and the average MUAs were calculated and smoothed by 5 or 10 ms sliding window averaging for each stimulation paradigm. For S1 and S2 evoked activity onset and duration, a threshold of baseline ±SD was set. For comparing S2 evoked activity in control versus TTX, the mean firing rate was calculated in two periods (0-100 ms and 100-1000 ms after onset of whisker deflection) for single whisker deflection. For whisker stimulation at 10 Hz, the mean firing rate was calculated in the time window after each whisker deflection.

#### Quantification of the fluorescent signal in wS1 and wS2 in histological samples

All image processing and quantification were performed in ImageJ. We selected the wS1 and wS2 in each image and calculated the mean fluorescent signal of each area. In addition, an area (in the hippocampus) which only has background signal but no strong thalamocortical fiber signal, was selected as a reference area. The wS1 and wS2 fluorescent signal was then normalized by subtracting the mean fluorescent signal to the reference area mean fluorescent signal. wS1/wS2 ratio was calculated with wS1 mean fluorescence divided by wS2 mean fluorescence in the same image without normalization.

#### Statistical analysis

The statistics are indicated for every experiment in the manuscript or figure legends. The Mann–Whitney test, paired *t* test, and one-way-ANOVA with Bonferroni's multiple comparison test were used to perform the statistical analyses.

## Results

### The spatiotemporal development of sensory-evoked input activity in wS1 and wS2

Research in adult mice has shown that the wS2 displays a topographic map of the whisker pad, albeit more compact and less well defined compared with the wS1 (Hubatz et al., 2020). Here, we performed simultaneous transcranial wide-field VSDI of wS1 and wS2 after deflecting individual whiskers of the contralateral whisker pad in four age groups across the first 2 weeks of postnatal (P) life under urethane-induced and maintained light anesthesia (Fig. 1; Extended Data Fig. 1-1). In all age groups, single whisker deflection led to the clear activation of two centers in the first 300 ms after the stimulus (Fig. 1*B*), with wS1 preceding wS2 (Fig. 1*C*). We found that before P5, wS1 and wS2 displayed activity that remained local for the duration of the signal, but starting at P6, the activity expanded beyond the two discrete points and merged into a large cortical area covering both. These results indicate that a rudimentary map of the whiskers is already present in wS2 during the first week after birth (Fig. 1*A*). It has been shown for wS1 responses that a clear map of the whiskers is also present at P3-P4, which is in agreement with the characteristic segregation of thalamocortical fibers and the formation of the barrels around P3 (Erzurumlu and Gaspar, 2012). These findings suggest that wS1 and wS2 are already functional early after birth and functionally segregated columns largely appear at the end of the first week.

10.1523/JNEUROSCI.2246-21.2022.f1-1 Figure 1-1 The onset times of evoked responses in wS1 and wS2, the duration at the half-maximal amplitude of VSDI in both regions, the evoked peak amplitude in the center of both regions and the wS1/wS2 ratios in different age groups (P1, P3, P6, and P14) are indicated in the tables. The values of mean and s.e.m for each group, the p values and t values of paired T test, the significance of one-way ANOVA with Bonferroni's multiple comparison test are also indicated in the tables. Download Figure 1-1, XLSX file.

With VSDI, a complex signal is recorded, which mostly arises from membrane voltage fluctuations of the cells because of synaptic activity (Chen et al., 2012). We first measured the duration at half-maximal amplitude for the two regions over time, which showed that the duration only significantly decreases in wS1 during the second postnatal week, while in wS2 the duration already becomes shorter at P6-P8 (Fig. 1*D*). This result may indicate that wS2 begins to receive faster synaptic inputs at the end of the first postnatal week, which would suggest an earlier maturation of its inputs compared with wS1. To assess the developmental time course of the strength of the inputs in the two cortical areas driven by whisker stimulation, we plotted the ΔF/F as a function of age for both regions independently. The data revealed that the overall activity generated in wS1 appears to reach a steady-state as early as P3 (Fig. 1*E*). In contrast, the maturation of incoming activity in wS2, compared with wS1, is delayed and reaches an upper plateau at P6-P8 (Fig. 1*F*). These results suggest that inputs to wS1 precede those to wS2. When plotting the ratio of activity between the two areas (wS1/wS2) across ages, we find that, at the end of the first postnatal week, inputs to both cortical areas become equally strong (Fig. 1*G*). This finding suggests that the amount of input-dependent excitation of wS1 and wS2 and the interaction between the two areas reaches a near stable state at the end of the first postnatal week.

### The spatiotemporal development of sensory-evoked output activity of wS1 and wS2 follows a bell-shaped curve

**Figure 2.**
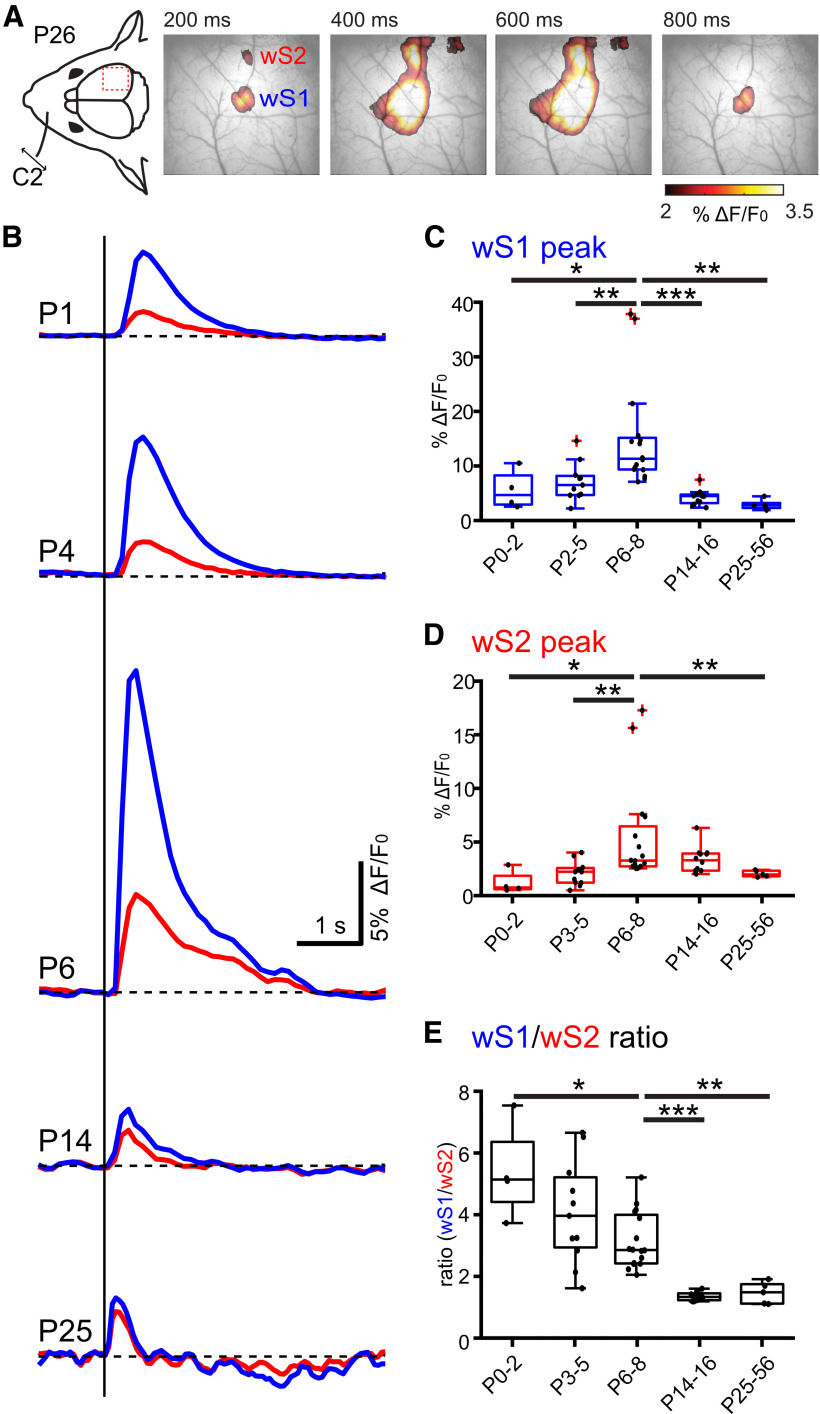
Developmental changes of sensory-evoked responses in wS1 and wS2 monitored with wide-field calcium imaging. ***A***, Left, Schematic illustration of the experimental design. A single whisker deflection is performed together with calcium imaging of the whisker somatosensory cortex of a P26 Snap25-GCamp6 mouse. Right, Evoked wide-field calcium imaging response at different time points after single C2 whisker deflection at 0 ms. Clearly separated responses in wS1 and wS2 are visible at 200 ms. ***B***, Temporal profile of wide-field calcium imaging responses recorded in the center of wS1 (blue trace) and wS2 (red trace) at different ages (P1, P4, P6, P14, and P25) following C2-whisker stimulation. ***C***, Box plot represents the evoked peak amplitude in the center of wS1 for different age groups (*N* = 4 P0-P2 mice; *N* = 11 P3-P5 mice; *N* = 16 P6-P8 mice; *N* = 11 P9-P12 mice; *N* = 5 P25-P56 mice). Statistics: Mann–Whitney test. P0-P2 versus P3-P5: *p* = 0.02; P3-P5 versus P6-P8: *p* = 0.002; P6-P8 versus P14-P16: *p* < 0.0001; P6-P8 versus P25-P56: *p* = 0.001. ***D***, Box plot represents the evoked peak amplitude in the center of wS2 for different age groups. Statistics: Mann–Whitney test. P0-P2 versus P6-P8: *p* = 0.0123; P3-P5 versus P6-P8: *p* = 0.0012; P6-P8 versus P25-P56: *p* = 0.0011. ***E***, Box plot represents the evoked peak amplitude in wS1 divided by the evoked peak amplitude in wS2 for different age groups. Statistics: Mann–Whitney test. P0-P2 versus P6-P8: *p* = 0.0206; P6-P8 versus P14-P16: *p* < 0.0001; P6-P8 versus P25-P56: *p* = 0.0011. In box plots, black dots represent the data points; red + signs indicate outliers. *p < 0.05; **p < 0.01; ***p < 0.001. All the values of data points and the mean and SEM of each group are in Extended Data Figure 2-1.

Having obtained a time course of synaptic input development across the two postnatal weeks for wS1 and wS2, we next sought to assess how the inputs may translate to an output. We therefore applied transcranial calcium imaging recording from mice expressing the genetically encoded calcium reporter (GCaMP6) in neurons under the Snap25 promoter (*Snap25-GCaMP6* mice), an approach that should primarily report action potentials. This allowed us to address the whisker-driven evoked responses with a mesoscopic spatial and temporal resolution of 5-15 µm and 100-200 ms, respectively. Similar to the VSDI data, after a single whisker deflection, the two activity spots in wS1 and wS2 were apparent in the first 200 ms of recording and merged over time in the adult (Fig. 2*A*). This experimental paradigm was performed in five age groups that spanned from the first postnatal day to 2-month-old lightly anesthetized mice (Fig. 2*B–E*; Extended Data Fig. 2-1). We found that the highest evoked calcium response was reached for both regions during the first postnatal week, but unlike the VSDI, the peak activity for both areas was reached maximally in the P6-P8 age group (Fig. 2*C*,*D*). When assessing the wS1/wS2 ratio of activity with age, we found that it is high in P0-P2 (5.36 ± 0.8, *n* = 4) and P3-P5 (4.03 ± 0.5, *n* = 11), and only starts to decrease at the P6-P8 time point (3.12 ± 0.23, *n* = 16), after which it gradually reduces at P14-P16 (1.3 ± 0.04, *n* = 12) and remains stable even at the P23-P56 age group (1.41 ± 0.16, *n* = 5). The wide-field calcium imaging results, together with the VSDI, reveal a window of increased input–output transformations taking place at the end of the first postnatal week for both wS1 and wS2.

10.1523/JNEUROSCI.2246-21.2022.f2-1 Figure 2-1 Evoked peak amplitude in the center of wS1 and wS2 for different age groups and wS1/wS2 ratio are indicated in the tables. The values of mean and s.e.m for each group, the p values of Mann–Whitney test are also indicated in the tables. Download Figure 2-1, XLSX file.

### The development of sensory-evoked spiking activity across layers in wS1 and wS2

**Figure 3.**
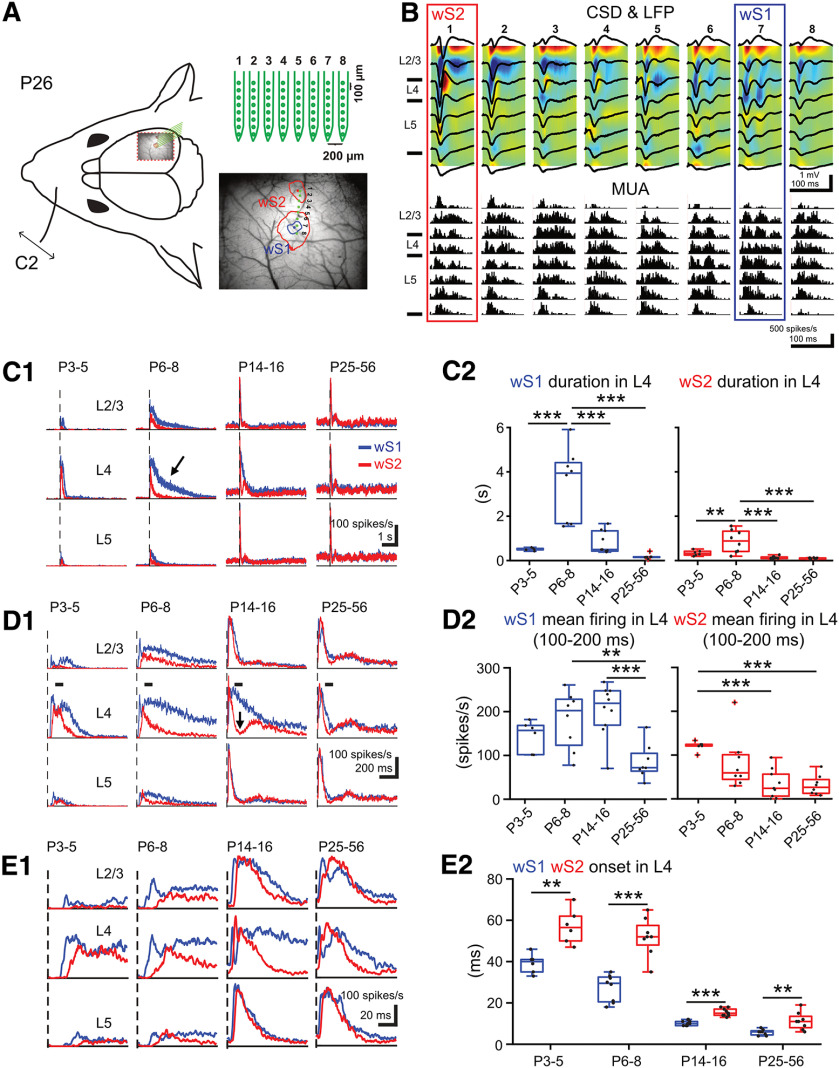
Developmental changes of sensory-evoked MUA in wS1 and wS2. ***A***, Left, Schematic illustration of the experimental design showing the insertion position of an 8 × 8 multielectrode probe array into wS1 and wS2 after identifying the center of evoked wS1 and wS2 by a single C2 whisker deflection in a Snap25-GCamp6 P26 mouse. Right, An example showing the evoked wide-field calcium imaging response (mean response from 11-40 ms after the onset of stimulation) in wS1 and wS2 by single C2 whisker deflection in a P26 mouse. We set 4% ΔF/F0 as the threshold to separate the evoked area of wS1 and wS2 (the red contour plot) and 5.5% ΔF/F0 as the threshold to confine the evoked area of wS1 (the blue contour plot). Blue dot indicates the center of evoked wS1. Red dot indicates the center of evoked S2. Eight green dots indicate the insertion location of the 8 × 8 multielectrode probe array. ***B***, Example of evoked local field potential (LFP) responses (black lines), color-coded CSD plots, and corresponding MUA responses elicited by a single C2 whisker deflection in a P26 mouse. The cortical layers (L2-3, L4, and L5) were identified by the evoked CSD pattern. ***C1***, Grand averages of evoked MUA responses recorded in L2-3, L4, and L5 of wS1 and wS2 (1 s before and 5 s after onset of stimulation) in 4 age groups (*n* = 6 in P3-P5, *n* = 8 in P6-P8, *n* = 10 in P14-P16, *n* = 8 in P25-P56). ***C2***, Box plots represent the duration of evoked MUA of wS1 and wS2 in L4 of the different age groups. Statistics: ANOVA with Bonferroni's multiple comparison test. S1: *p* < 0.0001; S2: *p* = 0.0004. ***D1***, Same as in ***C1***, but with higher temporal resolution showing only the first second after the onset of stimulation. ***D2***, Box plots represent the mean firing rate of L4 in wS1 and wS2 between 100 and 200 ms after stimulus in the different age groups. Statistics: ANOVA with Bonferroni's multiple comparison test. S1: *p* < 0.0001; S2: *p* = 0.0002. ***E1***, Same as in ***C1***, but with higher temporal resolution showing only the first 100 ms after stimulus. ***E2***, Box plots represent the evoked onset time of MUA in L4 of wS1 and wS2 for the different age groups. Statistics: paired *t* test. P3-P5: *p* = 0.0028, *t* = 5.47; P6-P8: *p* < 0.0001, *t* = 9.31; P14-P16: *p* < 0.0001, *t* = 10.11; P25-P56: *p* = 0.0017, *t* = 4.92. In box plots, black dots represent the data points; red + signs indicate outliers. *p < 0.05; **p < 0.01; ***p < 0.001. All the values of data points and the mean and SEM of each group are in Extended Data Figure 3-1.

**Figure 4.**
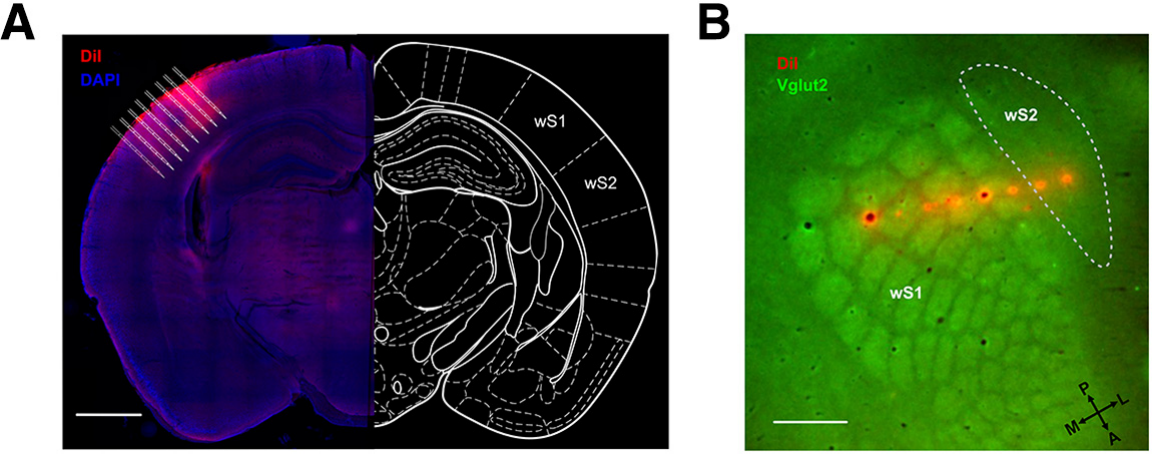
*Post hoc* anatomic confirmation of the silicon probe recording sites. ***A***, Example image reconstructing the recording tracks of the DiI labeled 8 shanks from the silicon probe wS1 and wS2 simultaneous recording from a P26 mouse. Scale bar, 1 mm. ***B***, Flattened cortical section reconstructing the recording sites of the DiI labeled 8 shanks from the silicon probe wS1 and wS2 simultaneous recording from a P41 mouse. Scale bar, 500 μm.

To directly examine the action potential generation of each cortical layer and its precise time course in wS1 and wS2 on whisker stimulation, we performed acute *in vivo* silicon probe recordings at the four key age groups identified with wide-field imaging (P3-P5, P6-P8, P14-P16, and P25-P56). We performed the same whisker deflection paradigm and recorded spiking activity across all layers in wS1 and wS2 simultaneously. In order to accurately locate the probe insertion region for wS2, the *Snap25-GCaMP6* mice were used, which allowed the identification of both areas and in relation to the blood vessel pattern. Subsequently, an 8 × 8 silicon probe array was inserted in such an orientation to record activity in both wS1 and wS2 at the same time (Figs. 3*A* and 4). By analyzing the local field potential and calculating the CSD profile, we were able to localize the recording sites along with the probes in respect to the cortical layers (Fig. 3*B*). We then extracted the MUA in the different layers for both regions across age. These analyses revealed significant developmental differences in the sensory activation of wS1 and wS2. The sensory evoked MUA pattern was short at P3-P5 (0.5 ± 0.02 s in wS1, 0.33 ± 0.05 s in wS2, *n* = 6), increased significantly at P6-P8 (3.44 ± 0.58 s in wS1, 0.87 ± 0.18 s in wS2, *n* = 8), and was short again in older age groups (Fig. 3*C*; Extended Data Fig. 3-1). These results are in line with the calcium wide-field imaging data, which also showed a developmental upregulation and downregulation of the output in wS1 and wS2 centered at the end of the first postnatal week (P6-P8). The analysis also showed that, at the end of the second postnatal week, layer 4 (L4) of wS2 displays a sharp decline of spiking activity (Fig. 3*D*), indicative of the emergence of feedforward inhibitory (FFI) control, potentially coming from the thalamic inputs driving wS2 L4, or even cortical input coming from wS1, which would developmentally engage more wS2 L4 through long-range connections. This result is unexpected since it could suggest that the higher-order area wS2 is regulated via developmental inhibitory control before primary wS1. The silicon probe data we obtained are in line with the calcium imaging results. The increased amplitude of the calcium imaging we observed at P6-P8 is underlined by a more prolonged spiking activity of the cortical circuit. In addition, these data also show that the spiking activity onset in wS1 precedes that in wS2 in all age groups (Fig. 3*E*; Extended Data Fig. 3-1). During the first postnatal week, the evoked onset times are long (39.2 ± 1.9 ms in P3-P5 wS1, 57 ± 4.4 ms in P3-P5 wS2, 27.2 ± 2.3 ms in P6-P8 wS1, 51.9 ± 3.3 ms in P6-P8 wS2), a finding that may also be because of the slow conduction velocity of the axons because of their incomplete myelination. Overall, these results demonstrate that wS2 is already strongly activated by sensory stimuli in the first postnatal week and undergoes more refined regulation during the second postnatal week.

10.1523/JNEUROSCI.2246-21.2022.f3-1 Figure 3-1 WS1 and wS2 L4 duration of evoked MUA in P3-P5, P6-P8, P14-P16 and P25-P56, both regions mean firing rate between 100-200ms (spikes/s) and both regions evoked onset times are indicated in the tables. The values of mean and s.e.m for each group, the significance of one-way ANOVA with Bonferroni's multiple comparison and p values and t values of the paired t test are also indicated in the table. Download Figure 3-1, XLSX file.

### Postnatal genetic disruption of thalamocortical inputs arrests the developmental progression of wS2 sensory-evoked activity

**Figure 5.**
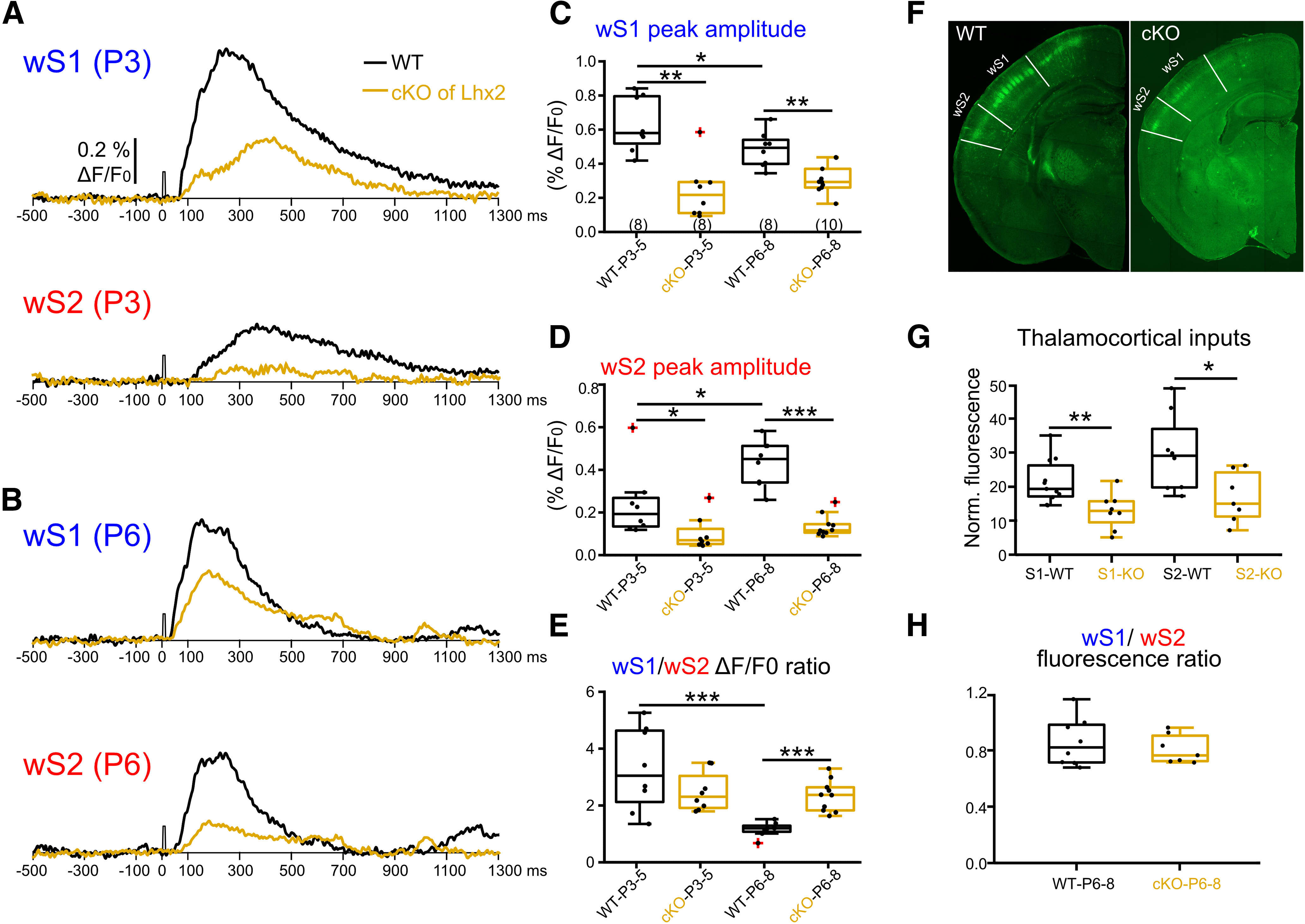
Postnatal removal of *Lhx2* from cortical excitatory cells leads to an arrest in the developmental progression of wS2 sensory-evoked activity. ***A***, An example trace of evoked VSDI recorded in the center of wS1 and wS2 from a P3 WT (black trace) and a P3 *Lhx2* cKO mouse (orange trace). ***B***, An example trace of evoked VSDI recorded in the center of wS1 and wS2 from a P6 WT and a P6 Lhx2 cKO mice. ***C***, Box plots represent the peak amplitude of evoked *VSDI* in wS1 of WT and cKO mice for the two age groups (*n* = 8 recordings from *N* = 4 P3-P4 WT mice; *n* = 8 recordings from *N* = 4 P3-P4 cKO mice; *n* = 8 recordings from *N* = 4 WT P6-P8 mice; *n* = 10 recordings from *N* = 6 P6-P8 cKO mice). Statistics: Mann–Whitney test. WT P3-P5 versus KO P3-P5: *p* = 0.0019; WT P6-P8 versus KO P6-P8: *p* = 0.0021; WT P3-P5 versus WT P6-P8: *p* = 0.0499. ***D***, Same as in ***C***, but for wS2. Statistics: Mann–Whitney test. WT P3-P5 versus KO P3-P5: *p* = 0.0207; WT P6-P8 versus KO P6-P8: *p* < 0.0001; WT P3-P5 versus WT P6-P8: *p* = 0.0148. ***E***, Box plots represent the evoked peak amplitude of VSDI in wS1 divided by the evoked peak VSDI amplitude in wS2 for the different age groups. Statistics: Mann–Whitney test. WT P3-P5 versus WT P6-P8: *p* = 0.0006; KO P3-P5 versus KO P6-P8: *p* = 0.63; WT P3-P5 versus KO P3-P5: *p* = 0.3823; WT P6-P8 versus KO P6-P8: *p* < 0.0001. ***F***, Example P7 brain images of Lhx2 WT (*Lhx2f/f:TCA-GFP*) and Lhx2 cKO (*Lhx2f/f:NexCre:TCA-GFP*) with wS1 and wS2 area indicated. ***G***, Box plots represent the normalized TCA-GFP fluorescent signal in wS1 and wS2 of WT and cKO mice at P6-P8 (*n* = 11 wS1 from *N* = 3 P6-P8 WT mice; *n* = 8 wS2 from *N* = 3 P6-P8 WT mice; *n* = 8 wS1 from *N* = 3 P6-P8 cKO mice; *n* = 7 wS2 from *N* = 3 P6-P8 cKO mice). Statistics: Mann–Whitney test. S1 WT versus S1 KO: *p* = 0.0073; S2 WT versus S2 KO: *p* = 0.0205. ***H***, Box plots represent the TCA-GFP fluorescence in wS1 divided by the TCA-GFP fluorescence in wS2 for both WT and cKO P6-P8 age group (*n* = 8 from *N* = 3 P6-P8 WT mice, *n* = 7 from *N* = 3 cKO mice). Statistics: Mann–Whitney test. *p* = 0.78. *p < 0.05; **p < 0.01; ***p < 0.001. All the values of data points and the mean and SEM of each group are in Extended Data Figure 5-1.

In order to assess the dependence of wS2 whisker-driven activity on direct thalamocortical inputs versus via wS1, we sought to disrupt thalamocortical inputs at the level of the cortex, but not the thalamus. To achieve this, we used a genetically modified mouse line in which the transcription factor *Lhx2* is floxed (Lhx2^fl/fl^) and postmitotically removed from cortical excitatory cells of all layers using the *Nex-Cre* mouse line (Goebbels et al., 2006). The loss of Lhx2 specifically in cortical neurons in the cKO (*Lhx2^fl/fl^:Nex-Cre*) was shown to disrupt thalamic projections to wS1 in the first postnatal week and the formation of the barrels, as well as lead to reduced whisker-evoked activation of the area (Shetty et al., 2013; Wang et al., 2017). In order to assess whether there is a similar disruption of thalamocortical projections in the cKO mouse line in both wS1 and wS2, we assessed when and how thalamocortical inputs may differentially affect the activation of wS1 and wS2 after whisker deflection, by performing concomitant VSDI in wS1 and wS2 in the cKO and WT for the Lhx2 allele mice. These experiments were performed in two age groups, P3-P5 and P6-P8. This choice is based on our previous functional experiments, which suggested that these age groups would represent direct thalamocortical activation only (P3-P5), in addition to the start of wS1-wS2 communication (P6-P8). The results showed that disrupting thalamocortical inputs to the somatosensory cortex in general reduces the sensory-evoked activity in wS1 and wS2, albeit in a differential manner. At P3-P5, there is a marginally stronger reduction in wS1 activation compared with wS2, whereas at P6-P8 the effect is much stronger on wS2 (Fig. 5*A–D*; Extended Data Fig. 5-1). This is also reflected in the ratio of activation of wS1/wS2, which is not statistically different in the cKO mice compared with their littermate controls at P3-P5, but it becomes significantly higher in cKO at P6-P8 (1.16 ± 0.09 for WT vs 2.33 ± 0.17 for KO, *p* < 0.0001, Fig. 5*E*). To test whether wS1 and wS2 have a similar level of thalamocortical input disruption in the Lhx2 cKO at P6-P8, we crossed the cKO to a mouse line that labels VPM axons with GFP (*TCA-GFP*) (Mizuno et al., 2014) to quantify the intensity of fluorescent signals of GFP-positive thalamocortical fibers in control versus cKO in wS1 and wS2 (Fig. 5*F–H*). We found that both wS1 and wS2 had significantly decreased fluorescence in the Lhx2 cKO compared with WT animals (Fig. 5*G*), and that the wS1/wS2 ratio did not differ between WT and Lhx2 cKO at P6-P8 (Fig. 5*H*). Notwithstanding the caveats of not assessing POm axons, our data suggest that wS2 is functionally more disrupted on whisker stimulation in the Lhx2 cKO despite the similar thalamocortical (VPM) disruption in wS1 and wS2. This finding corroborates the hypothesis that, by P6-P8, the activation of wS2 after whisker deflection is a summation of both direct thalamic input and excitation coming from wS1, hence the stronger relative reduction of the overall activity of wS2 in this age group.

10.1523/JNEUROSCI.2246-21.2022.f5-1 Figure 5-1 Peak amplitude of VSDI in wS1,wS2 and wS1/wS2 ratio, P7 thalamocortical fluorescent signals in wS1 and wS2 and their ratio values are indicated in the table. The values of mean and s.e.m for each group, the p values of the Mann–Whitney test are also indicated in the table. Download Figure 5-1, XLSX file.

### Acute blockade of wS1 activity identifies a developmental window for information transfer between wS1 and wS2

**Figure 6.**
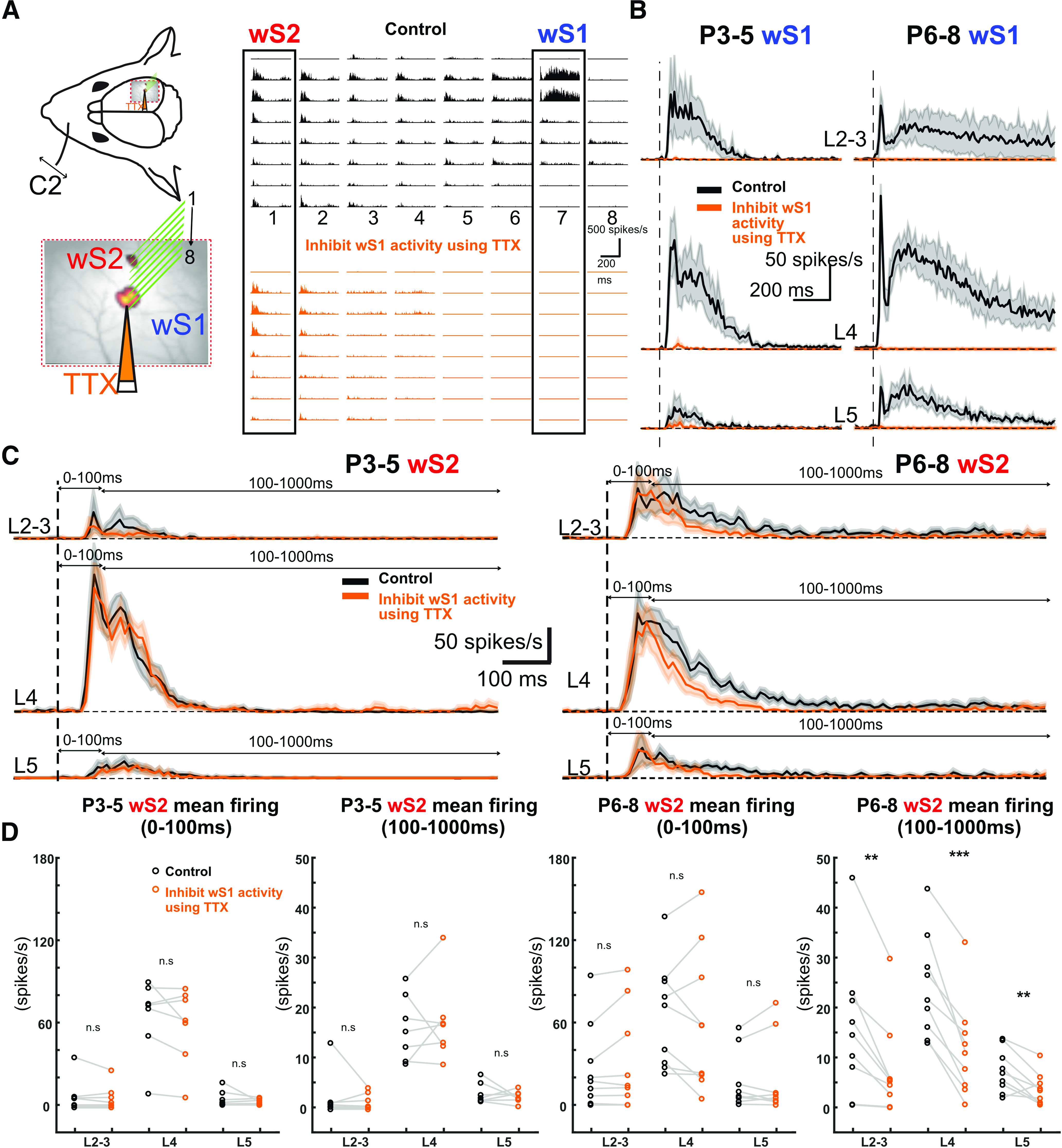
Acute inhibition of wS1 activity differently influences sensory-evoked responses of S2 over development. ***A***, Left, A schematic illustration showing the insertion position of an 8 × 8 silicon probe array in wS1 and wS2. TTX was injected into wS1 through a micro-glass pipette (orange). Right, An example of evoked MUA recorded in wS1 and wS2 after C2 whisker deflection in a P8 mouse in the control condition (black) and after local TTX injection in wS1 (orange). ***B***, Average traces of evoked wS1 MUA in L2-3, L4, and L5 of the P3-P5 age group (left, *n* = 7) and P6-P8 age group (right, *n* = 9). Black trace represents before injecting the TTX (control). Orange trace represents TTX injection. Shaded areas represent SEM. ***C***, Average traces of evoked wS2 MUA in L2-3, L4, and L5 of the P3-P5 age group (left, *n* = 7) and P6-P8 age group (right, *n* = 9). Black trace represents before injecting the TTX (control). Orange trace represents TTX injection. Shaded areas represent SEM. ***D***, wS2 evoked MUA mean firing rate (spike/s) in L2-3, L4, and L5 for the time windows of the first 100 and 100-1000 ms in P3-P5 (*n* = 7) and P6-P8 (*n* = 9) age groups. Black represents before TTX injection in wS1. Orange represents after TTX injection in wS1. Statistical comparison was performed between control and TTX injection conditions using the paired *t* test for both time windows, both age groups, and all the layers: **p < 0.05; ***p < 0.001. P6-P8 wS2 100-1000 ms L2: *p* = 0.004, *t* = 3.99; P6-P8 wS2 100-1000 ms L4: *p* < 0.0001, *t* = 7.22; P6-P8 wS2 100-1000 ms L5: *p* = 0.009, *t* = 3.41. All the values of data points, the mean and SEM of each group, and statistical *p* and *t* values are in Extended Data Figure 6-1.

**Figure 7.**
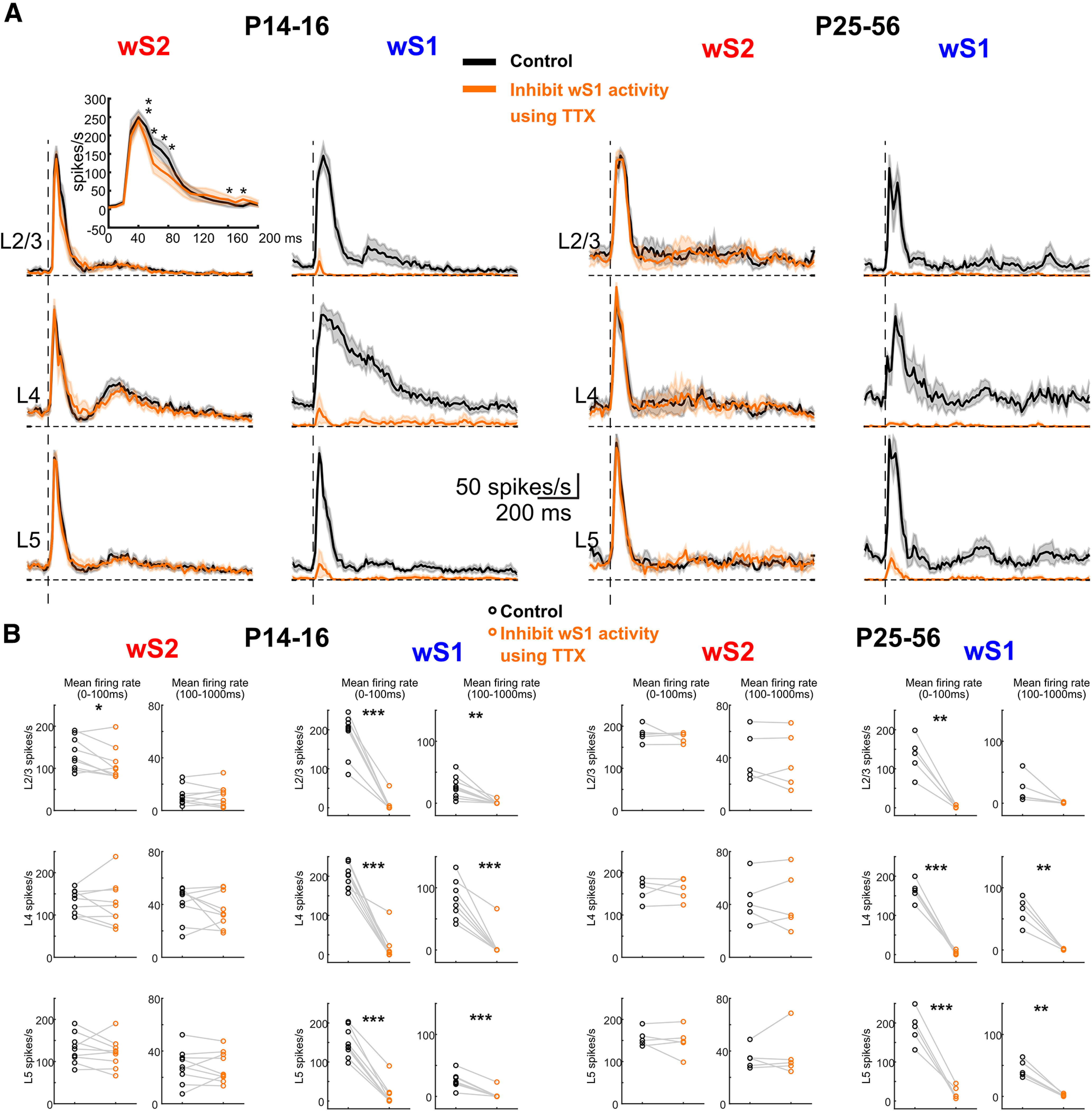
Inhibiting S1 influences the sensory-evoked response of S2 single whisker deflection. ***A***, Left, Average of wS2 and wS1 evoked MUA response in L2-3, L4, and L5 of P14-P16 age group mice (*n* = 9) in the control condition (black) and TTX injection in wS1 (orange). Inset, The detailed paired *t* test comparison of mean firing rate between control and TTX injection conditions using 10 ms bins during the period of 0-200 ms after the onset of whisker stimulation. Right, Average of wS2 and wS1 evoked MUA response in L2-3, L4, and L5 of P25-P56 age group mice (*n* = 5) in the control condition (black) and TTX injection in S1 (orange). ***B***, The mean firing rate of wS2 and wS1 evoked response in L2-3, L4, and L5 during the period of 0-100 and 100-1000 ms after the onset of the whisker stimulation of P14-P16 and P25-P56 age groups in the control (black) and TTX injection in S1 (orange) condition. Statistical comparison was performed between control and TTX conditions: *p < 0.05; **p < 0.01; ***p < 0.001; paired *t* test. All the values of data points, the mean and SEM of each group, and statistical *p* and *t* values are in Extended Data Figure 7-1.

**Figure 8.**
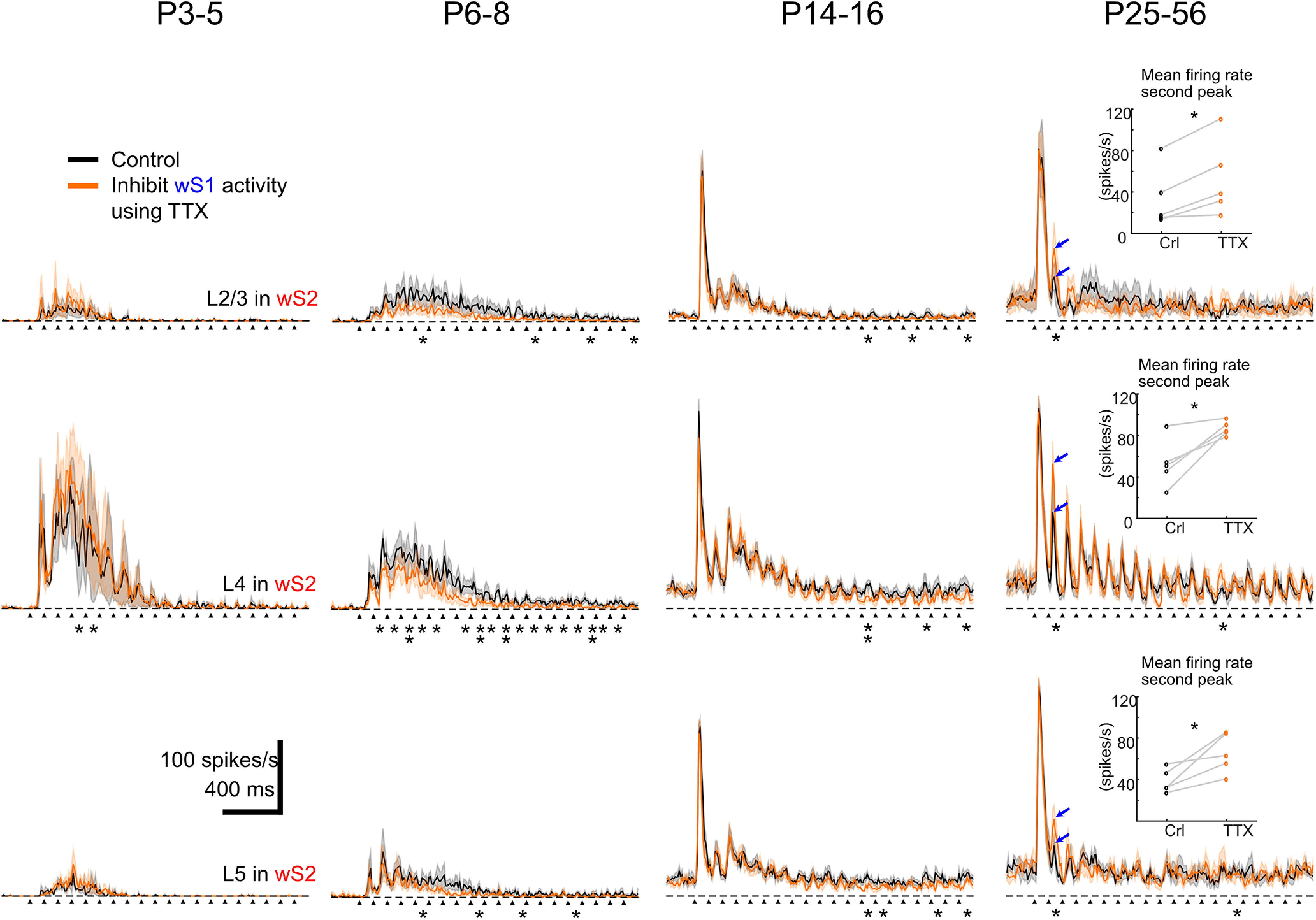
Inhibiting S1 influences the sensory-evoked response of S2 by single whisker deflection at 10 Hz for 2 s. Average of evoked MUA response in L2-3, L4, and L5 of P3-P5 (*n* = 3), P6-P8 (*n* = 6), P14-P16 (*n* = 7), and P25-P56 (*n* = 5) age groups of mice. Black arrows indicate the onset of whisker deflection. Asterisks indicate significant difference between control and TTX injection conditions using the paired *t* test during the period of 0-100 ms after the onset of each whisker stimulation. Inserted plots at P25-P56 are the mean firing rate during the period of 0-100 ms after the onset of the second whisker stimulation at L2-3, L4, and L5. Blue arrows indicate the peaks of averaged MUA response after the second whisker stimulation. Statistical comparison was performed between control and TTX injection conditions using the paired *t* test for all the 100 ms time windows. *p < 0.05. **p < 0.01. All the values of data points and statistical *p* values are in Extended Data Figure 8-1.

It is known that, in the adult mouse cortex, wS1 sends projections to wS2 and vice versa (El-Boustani et al., 2020). To assess whether the changes that we observe in wS1 and wS2 VSDI between P3-P5 and P6-P8 are driven by the propagation of activity from the former to the latter, we performed another set of silicon probe experiments where we acutely silenced action potential firing in wS1 and assessed spiking changes in wS2 after whisker deflection. We first tested whether the application of TTX was able to silence the underlying activity in wS1 *in vivo*. Indeed, when recording spontaneous spiking in wS1, as well as whisker-evoked activity, we saw no action potentials occurring in the presence of TTX, indicating that this manipulation was efficient in abolishing activity for all the age groups on which we focused: P3-P5, P6-P8, P14-P16, and P26-P46 (Figs. 6*A*,*B* and 7). In contrast, analysis of the activity in wS2 after silencing wS1 showed no apparent effect in wS2 at P3-P5 (Fig. 6*C*,*D*; Extended Data Fig. 6-1). Nevertheless, there was a significant decline of wS2 spiking activity in the P6-P8 age group in all layers when analyzing the 100-1000 ms time window after the onset of whisker stimulation (Fig. 6*C*,*D*). This result indicates that both wS1 and wS2 received thalamocortical inputs from P3 onward and that wS2 starts receiving excitatory input from wS1 from P6 onward. The effect of the spiking activity was also observed at P14-P16, but it was only present in L2/3. Specifically, in the 50-80 ms time window after onset of whisker stimulation, there was a reduction of the spiking activity, while there was a slight increase in the 160-180 ms window (Fig. 7*A*; Extended Data Fig. 7-1). Notably, the sharp decline of the spiking activity in wS2 L4 was still present after silencing wS1, which indicates that the feedforward inhibition wS2 L4 received at P14-P16 is not triggered by wS1 inputs. In adolescent/adult animals, abolishing activity in wS1 lead to no observable difference in wS2 with a single whisker deflection. Since the mouse starts to whisk at high frequency around P14 (Landers and Zeigler, 2006; Arakawa and Erzurumlu, 2015), a 10 Hz single-whisker stimulation was also used to simulate the high-frequency contact for all age groups and the same wS1-silencing experiment was performed. We found that the P6-P8 age group had general decreased evoked MUA activity in wS2 L4 from the second whisker stimulus onward, while the P3-P5 and P14-P16 age groups did not present significant changes before being activated by several whisker stimuli (Fig. 8; Extended Data Fig. 8-1). However, an increased second peak of wS2 activity was observed after the second deflection during the 10 Hz whisker stimulation in the P25-P56 age group (Fig. 8; Extended Data Fig. 8-1). This result suggests that the excitatory influence of wS1 onto wS2 goes through a refined regulation after the end of the second postnatal week, which may be because of the maturation of inhibitory components being activated in wS2. These findings support the calcium imaging and VSDI data in control and cKO mice and point at a developmental window for excitatory information transfer from wS1 to wS2 at the end of the first and beginning of the second postnatal week, indicating the emergence of higher-order cortical processing of sensory information. In the third postnatal week and coinciding with the onset of active exploratory whisking behavior, this excitatory drive of wS2 by wS1 is not apparent, and rather inhibition may also be recruited. This could represent an extra level of regulation of the information necessary to build proper sensory representations in wS2.

10.1523/JNEUROSCI.2246-21.2022.f6-1 Figure 6-1 The mean firing rate of wS2 and wS1 evoked response in L2-3, L4, and L5 during the period of 0-100ms and 100-1000ms after the onset of the whisker stimulation of P3-P5 and P6-P8 age groups in the control and TTX injection in S1 condition. Paired t test was used to compare between control and TTX for 0-100ms and 100-1000ms. Mean, s.e.m., p values and t values are indicated in the table below. Download Figure 6-1, XLSX file.

10.1523/JNEUROSCI.2246-21.2022.f7-1 Figure 7-1 P16 and P25-P56 age groups in the control and TTX injection in S1 condition. Paired t test was used to compare between control and TTX for 0-100ms and 100-1000ms. Mean, s.e.m., p values and t values are indicated in the table below. P14-P16 detailed analysis for every 10ms is shown in the lower table. P values are also indicated by using a paired T test comparing control and TTX for every 10ms after the onset of whisker deflection. Download Figure 7-1, XLSX file.

10.1523/JNEUROSCI.2246-21.2022.f8-1 Figure 8-1 wS2 evoked MUA mean firing rate (spike/s) for every 100ms after the onset of whisker deflection in L2-3, L4, and L5 of P3-P5, P6-P8, P14-P16. Paired T test was used to compare between control and TTX for every 100ms after the onset of whisker deflection. P values are indicated in the table below. One star (*) indicates p < 0.05 and two stars (**) indicate p < 0.01. Download Figure 8-1, XLSX file.

**Figure 9.**
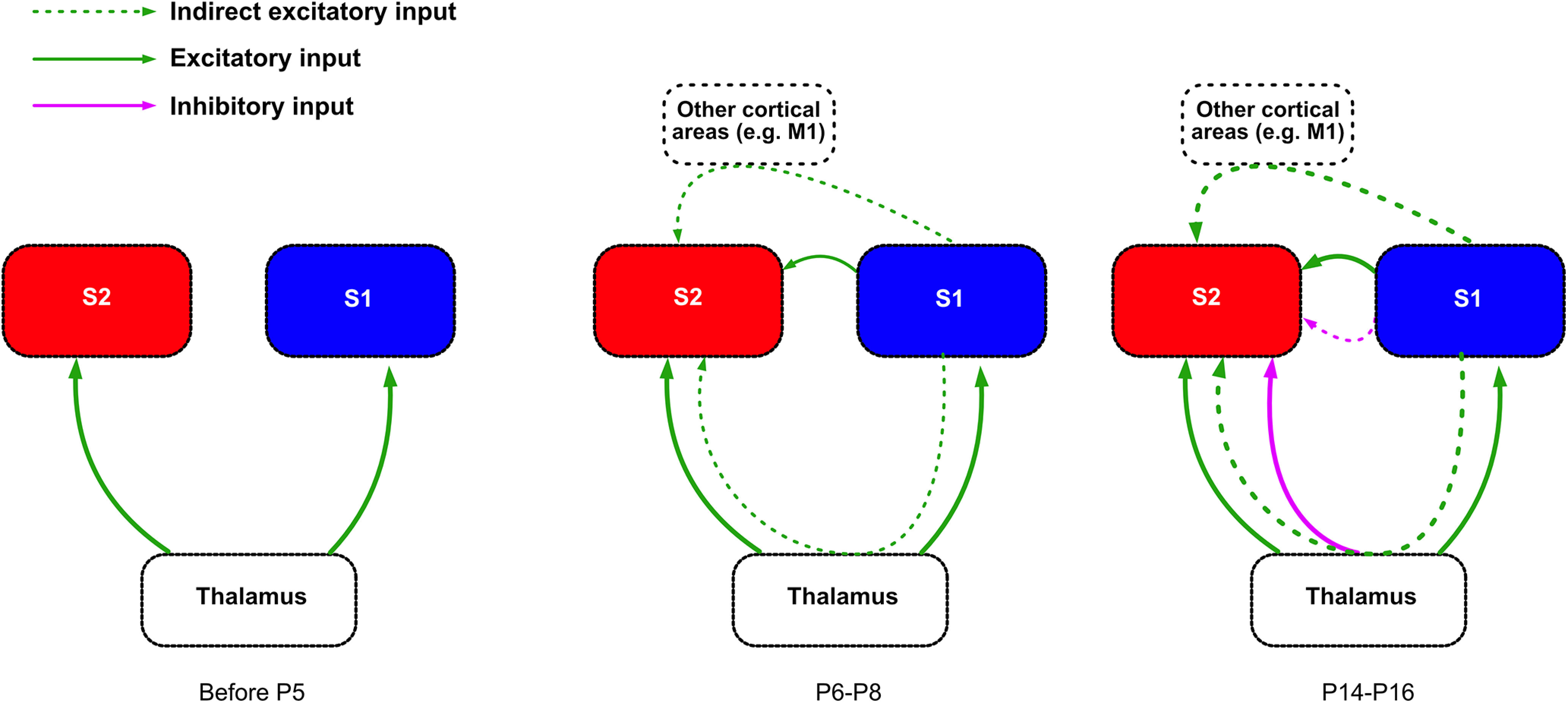
Schematic of the developmental functional connectivity between wS1 and wS2. Thalamocortical input: Both wS1 and wS2 already receive input from the thalamus shortly after birth, and this input becomes stronger during the second postnatal week. Corticocortical connectivity: Before P5, L2-3 of wS1 and wS2 receive very little input from the respective L4, and functional connectivity from wS1 to wS2 is not yet developed. At P6-P8, L2-3 in both wS1 and wS2 receive more activation on whisker stimulation, and wS2 L2-3 and L4 start receiving inputs from wS1. At the end of the second postnatal week, wS2 starts receiving inhibitory regulation from either the thalamus or wS1 or both while the excitatory inputs mature.

Overall, our data identify a developmental time window in which activity from wS1 starts impacting that of wS2 on the presentation of a sensory stimulus. Although before P5 both wS1 and wS2 already receive functional thalamocortical inputs, there is little communication between the two cortical areas (Fig. 9). It is only at the end of the first postnatal week that wS2 starts to receive excitation from wS1, which can be direct between the two sensory areas via corticocortical connections or also indirect through the thalamus or even other brain regions, such as the primary motor cortex (M1). The communication between wS1 and wS2 becomes more refined during the second postnatal week, possibly because of the appearance of inhibition within and in between these two areas (Fig. 9).

## Discussion

In this study, we examined when wS2 begins to be developmentally activated by whisker deflections and how wS1 begins communicating with wS2 postnatally, to assess when higher-order processing of whisker stimuli commences.

By using two wide-field imaging approaches, one of which provides a readout of the synaptic inputs a region receives (VSDI), whereas the other a readout of spiking (calcium imaging), we identified the developmental time course of input–output transformations in wS1 and wS2. VSDI indicated that the incoming activation on whisker stimulation in wS1 matures a few days before wS2, and is already at steady state by postnatal day 3 (P3). In contrast, imaging from all neurons in the two regions, revealed that their output is more synchronized and tracks with the increased inputs that wS1 receives. Our follow-up silicon probe-based electrophysiological recordings in wS1 and wS2 consolidated the calcium imaging data and provided insights into the temporal domain of spiking activity across cortical layers. The results show that, during the first postnatal week, whisker deflection leads to a faster onset of wS1 spiking activity compared with wS2, a difference that gets reduced with time and reaches its lowest point in adult mice. The recordings also reveal that there is an increase in the sustained spiking activity of the two regions at P6-P8, albeit more so in the wS1 compared with wS2. This time window coincides well with the increase in the generation of lateral excitatory connectivity between pyramidal cells of L2/3 in wS1 (Bureau et al., 2004), thereby providing an underlying circuit-based explanation for the large increase in sustained activity in wS1, which is then transferred to wS2 after a whisker stimulus. We also find that the sustained excitability of layer 4 in wS2 at P6-P8 is curtailed at P14-P16 much more strongly than in wS1. This finding suggests that a type of sensory-driven FFI control of wS2 develops earlier than wS1, the latter only showing the same pattern in layer 4 after P26. As a higher-order cortical area, it is expected that FFI in wS2 would lag behind. The presence of early FFI in wS2 in our data would suggest an earlier maturation of certain aspects of signal processing in wS2 compared with wS1, the validation and mechanism of which require further experimentation.

To assess the direct thalamocortical activation of wS1 and wS2, versus from the former to the latter, we took advantage of a genetically modified mouse line that displays disrupted thalamocortical projections upon the removal of the transcription factor Lhx2 from cortical excitatory neurons (*Lhx2f/f; Nex-Cre*). In these mice, the thalamus remains genetically unchanged while the cortex is affected, including the somatosensory and visual cortices (Zembrzycki et al., 2015; Wang et al., 2017). By using the *Lhx2^fl/fl^:Nex-Cre:TCA-GFP* mouse line and quantifying the wS1 and wS2 TCA-originating GFP fluorescence, we found that the disruption of the thalamocortical inputs in this mouse line is of the same magnitude for both wS1 and wS2 (Fig. 5*F*,*G*). The TCA-GFP reporter has been shown to label sensory thalamus, and fluorescent signal is seen early postnatally in both wS1 and wS2 (Mizuno et al., 2014). We propose that the difference we observe in the activation ratio of the two areas in this mouse line after whisker stimulation is attributed to defects in additional activity coming from other cortical areas. Indeed, we observed a much more pronounced reduction of wS2 activity compared with wS1 at P6-P8 in cKO mice, but not at P3-P4 (Fig. 5*D*). Our results support that, initially, the thalamocortical inputs are equally disrupted, whereas a few days later, wS2 has also “lost” the indirect sensory-driven activity that drives it further via wS1. In line with this, we observe that in WT mice the wS1/wS2 ratio decreases from P3-P5 to P6-P8 (Figs. 1*G* and 5*E*) because of the increased activity in wS2 (Figs. 1*F* and 5*F*) provided by the extra input from wS1 to wS2 at P6-P8. However, it should be noted that our analysis on the *Lhx2^fl/fl^:Nex-Cre:TCA-GFP* mouse line does not allow us to assess the contribution of the thalamocortical inputs arising from POm versus the VPM on our phenotype. To begin with, it is unknown whether both wS1 and wS2 are mainly driven by VPM at the young age. In addition, more experiments would be needed to investigate whether the genetic manipulation has an effect on the trans-laminar cortical activity within wS1 and wS2. It is therefore possible that our results on these mouse lines could also be explained by a differential developmental defect in POm versus VPM inputs to wS1 and wS2 and/or by differential alteration of the local cortical circuit of the two areas. Despite the caveats, our results on the genetically modified mice suggested that the corticocortical communication from wS1 to wS2 could affect the activation of wS2. Therefore, we directly tested the contribution of wS1 activity to sensory-driven activation of wS2 by inhibiting action potential firing in wS1. We find that this acute activity manipulation at P6-P8 reduces the activity in all layers of wS2, with the larger difference observed in L4. The layer dependency of this effect matches to a large extent the anatomic axonal innervation that has been reported in adult mice between wS1 and wS2, appearing in more or less all layers of wS2 and also heavily innervating L4 (Minamisawa et al., 2018). Interestingly, the innervation between L4 cells of the two areas may be even more pronounced at P6-P8, since it has been observed that L4 wS1 axons from stellate cells send long-range projections contralaterally that subsequently trim before the end of the second postnatal week (De León Reyes et al., 2019).

In line with the wide-field results, before P6-P8, we observed no change in the spiking of wS2 after acutely blocking activity in wS1. A few days later, at P6-P8, we saw that information is fed forward from wS1 to wS2, as indicated by the reduction of the late spiking activity of wS2, when abolishing activity in wS1. At P14-P16, blocking wS1 activity decreases spiking within 50-80 ms in L2/3 followed by a slight increase of spiking at 160-180 ms. This bivalent effect at P14-P16 seems to suggest that this period is a transition one, since after that (>P26), this effect is not observed. In contrast, an overall inhibitory influence of wS1 onto wS2 was revealed on repetitive deflection of the whiskers at 10 Hz, which is in line with a known temporal bias in information transfer to wS2 (Melzer et al., 2006) and matches the whisking frequency of adult animals (Landers and Zeigler, 2006; Arakawa and Erzurumlu, 2015). This finding in adult animals is intriguing, as it is generally considered that wS1 would transmit information to wS2 in a positive feedforward manner. Although this is not what we find, it may be the case when animals are performing a behavioral tactile-dependent task (Chen et al., 2015), despite the fact that to our knowledge inactivating wS1 and assessing the activity in wS2 has never been directly tested in rodents. Nevertheless, there is evidence in cats, rabbits, and marmosets that supports a parallel processing model of information coming directly from the thalamus, compared with a sequential wS1 to wS2 one (Manzoni et al., 1979; Burton and Robinson, 1987; Turman et al., 1995; Zhang et al., 1996). These studies have shown that a large number of neurons in wS2 showed no change in their sensory-driven activation when wS1 was acutely, and in some cases reversibly, inactivated, whereas others display a small decrease. Based on these findings, more functional experiments are required in awake behaving mice to determine the direct impact of wS1 activity to wS2 in adulthood. Overall, our data show that the functional communication between wS1 and wS2 begins at the end of the first postnatal week and develops in a more complex manner the following weeks. Although we would propose that this communication is direct between the two areas and supported by anatomic connections that seem to be established around P6-P8 (Klingler et al., 2021), the methodology that we have applied herein cannot exclude other indirect pathways of communication. These would include pathways from wS1 to wS2 through wM1 or the thalamus, especially during the late spiking phase. Interestingly, published work has provided some insights into the development of parallel versus sequential activation between somatosensory paw area S1 (pS1) and paw primary motor cortex (pM1) of rat (Gómez et al., 2021).

In conclusion, our data provide novel insights into the sensory-driven developmental activation of a secondary somatosensory area and the transfer of information into it from the respective primary area. We have revealed a previously unidentified developmental time window for the positive information flow from wS1 and wS2 at the end of the first postnatal week, which could be suggestive of the beginning of more complex representations of the sensory environment in the cortex before active exploration begins.
